# Correction: Using imaging photoplethysmography for heart rate estimation in non-human primates

**DOI:** 10.1371/journal.pone.0211518

**Published:** 2019-01-25

**Authors:** Anton M. Unakafov, Sebastian Möller, Igor Kagan, Alexander Gail, Stefan Treue, Fred Wolf

The following information is missing from the Funding section: This study was supported by the German Research Foundation and the Open Access Publication Funds of the Göttingen University.
