# Supplementary figures and images for: Using imaging photoplethysmography for heart rate estimation in non-human primates
Source: PLoS One. 2018 Aug 31;13(8):e0202581. doi: 10.1371/journal.pone.0202581 (PMC6118383; doi:10.1371/journal.pone.0202581)

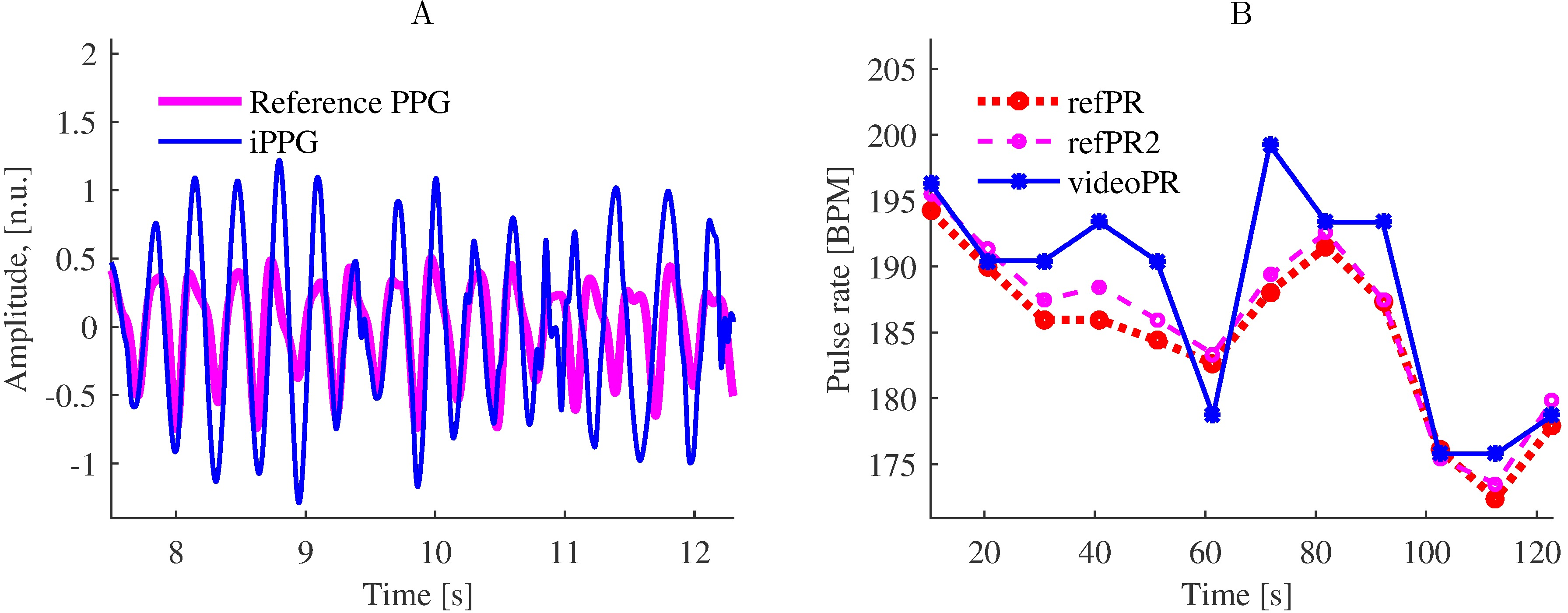

Supplement: S1 Fig — (A) Imaging photoplethysmogram (iPPG) aligns with contact photoplethysmogram (PPG) recorded using pulse oximeter P-OX100L (Medlab GmbH, Stutensee; documented accuracy ± 1%) for Session 8 (in addition to the basic reference pulse oximetry, which was the same as for other sessions). (B) Pulse rate estimated from this more precise PPG (refPR2) has very good agreement with the reference pulse rate refPR (mean absolute error 1.41, Pearson correlation 0.98). Notably, videoPR computed from iPPG has even better agreement with refPR2 than with refPR (mean absolute error 3.24, Pearson correlation 0.90). Comparison of imaging photoplethysmogram with a contact photoplethysmogram. (A) Imaging photoplethysmogram (iPPG) aligns with contact photoplethysmogram (PPG) recorded using pulse oximeter P-OX100L (Medlab GmbH, Stutensee; documented accuracy ± 1%) for Session 8 (in addition to the basic reference pulse oximetry, which was the same as for other sessions). (B) Pulse rate estimated from this more precise PPG (refPR2) has very good agreement with the reference pulse rate refPR used in all sessions (mean absolute error 1.28 BPM, Pearson correlation 0.99). Notably, videoPR computed from iPPG has even better agreement with refPR2 than with refPR (mean absolute error 3.24 BPM, Pearson correlation 0.90, cf. metrics of correspondence for videoPR and refPR in Table 5). (TIF) [file pone.0202581.s001.tif]
